# Maximizing economic prosperity, strengthening military, or developing social bonds? Study protocol for research on the relationship between regulatory focus and preferences for the direction of societal development

**DOI:** 10.1371/journal.pone.0274624

**Published:** 2022-09-26

**Authors:** Marta Roczniewska, Magdalena Marszałek, Kamila Kaffka-Skierka, Kuba Kryś, Agata Gasiorowska

**Affiliations:** 1 SWPS University of Social Sciences and Humanities, Institute of Psychology, Warsaw, Poland; 2 Department of Learning, Informatics, Management, and Ethics, Karolinska Institutet, Stockholm, Sweden; 3 Institute of Psychology, Polish Academy of Sciences, Warsaw, Poland; Public Library of Science, UNITED STATES

## Abstract

Recent research has pointed to cross-cultural differences with regard to preferences for the directions that societies should take in their development. From an individual’s perspective, these directions might be understood as ‘goals’, i.e., internal representations of desired end states. To date, research on individual differences that determine preferences for such directions is scarce. However, people’s motivational concerns, i.e., what they fundamentally value, may shape their views about the desired paths for their country’s future. The role of such motivational concerns has been described by regulatory focus theory, which distinguishes between promotion concerns related to advancement needs and prevention concerns linked with security needs. The overall aim of this project is to map the different pathways of societal development with regulatory focus concerns. This will be achieved in two studies. In Study 1, a group concept mapping method will be employed, and leading psychologists will assess the extent to which various societal development goals represent promotion and prevention goals. Based on these ratings, a two-dimensional map of the goals will be created and presented to the same experts, who will be asked to create goal clusters based on their proximity with regard to promotion and prevention ratings. This study will reveal which societal development directions have promotion concerns that outweigh prevention concerns (and vice versa) and which are both high (or low) on these dimensions. This initial mapping will be corroborated in correlational Study 2 with representative samples from two countries differing in dominating regulatory orientations (Poland vs. USA). Here, the roles of individual promotion and prevention orientations in preferences for specific societal development directions will be evaluated. This project will provide new insights into the roles of individual motivational systems in preferences for goals that might be pursued in country development.

## Introduction

For years, economic growth has been equated with the development of a society [1]. However, more recently, societies have begun to question the direction in which they should develop. Keeping economic priority high is understandable for less prosperous societies because it brings significant improvements in quality of life [2,3]. It could be argued, however, that in several countries in the contemporary world, the basic needs of most citizens are satisfied, and therefore, these countries can be considered economically prosperous enough to focus on societal goals beyond economic expansion [4]. For instance, an alliance of several countries has recently launched a Wellbeing Economy Governments (WEGo) group where member countries are working together to understand the key priorities for their citizens’ wellbeing. Cross-cultural research has shown that economic growth is only one of many directions of societal development. However, people tend to give little support to the conventional aims of societal development, such as pumping up the military, demographic characteristics, or upholding cultural traditions. Much stronger and universal across cultures is the expectation to prioritize modernization in its various facets: from the fundamentals of modernization (e.g., fostering trust, social bonds, and safety), to welfare aims (e.g., education, justice, and poverty eradication), and to inclusivity goals (e.g., gender equality, human rights, openness to new people and ideas) [5]. Previous research paved the pathway to studies on the cultural sensitivity approach to this matter, examining societal development from a macrosocial perspective (i.e., describing cultural similarities and differences). The individual level of analysis, which answers the question of what guides individuals to prefer different types of societal development, remains understudied. Here, this gap in research is addressed.

From an individual perspective, the directions of societal development could be understood as *goals*, that is, internal representations of desired states in a given domain [6]. To date, however, research has lacked a bottom-up approach that focuses on the various ways in which people’s individual motivational concerns (i.e., their needs and core values) shape their views about the desired directions for their country’s progress. We propose that to understand what directions countries should take in their development from the perspective of their citizens, it is critical to understand the motivational systems that underlie both the selection and pursuit of goals.

One framework that offers potential for such insights is the regulatory focus theory (RFT; [7]). The RFT assumes that people have two independent motivational systems that are grounded in distinct fundamental needs. The RFT distinguishes between promotion concerns related to advancement needs (e.g., growth or development) and prevention concerns linked with security needs (e.g., safety and protection). These concerns determine the selection and pursuit of goals, which is reflected in the desired end states that people value and in their preferred strategies and tactics to reach those goals [8]. Thus, we believe that the chronic self-regulatory orientation, as described by the RFT, may explain differences in individual preferences for the country’s developmental directions.

In sum, the overall goal of this project is to examine different paths of societal development from the perspective of the RFT. In this way, understanding will be gained with regard to whether certain goals are associated with fundamentally different concerns (growth vs. safety) and whether their importance may depend on individual differences in motivational systems related to these concerns. This is important because, while it is assumed that the structures of society are responsible for shaping individuals, there are individual variations within the macro-cultural context due to individual differences that could determine which goals certain people would support. For example, previous research has shown that a regulatory focus is a better predictor of achievement-related behaviors than the cultural dimensions of collectivism versus individualism and that it mediates the relationship between these two dimensions and participants’ behaviors [9]. While regulatory focus may be triggered by situational cues [10,11], there is already strong evidence for chronic differences in regulatory focus [12–14]. In addition, cross-cultural studies [9,15,16] have shown that certain cultures are characterized by the prevalence of a particular regulatory focus among their members. The main claim of the present article is that chronic regulatory foci should be considered as explanatory factors for the cross-cultural differences in preferred societal development directions.

### Regulatory focus theory

The RFT [7] distinguishes between two systems of goal pursuit: promotion and prevention. These systems are grounded in different sets of basic human needs. The *promotion* system originates from the needs for growth and advancement. Promotion-focused individuals are oriented toward gains: they want to move from the current status quo “0” to a better state “+1”. Consequently, individuals with a promotion focus are primarily concerned with the presence or absence of positive end states. They also show positive implicit attitudes toward objects that bring them closer to desired end states [17,18], and they prefer eager strategies in their decision-making and goal pursuit [19,20]. Promotion-focused individuals represent goals as ideals, hopes, and aspirations. In turn, the *prevention* system is rooted in safety and security needs [7]. Prevention-focused individuals are motivated to maintain the satisfactory status quo versus a worse “-1” end-state. Thus, people with a prevention focus are primarily concerned with the absence or presence of negative end states (non-losses vs. losses). Therefore, individuals with a high prevention focus prefer vigilant strategies and error monitoring in their decision-making and goal pursuit [19,20]. Moreover, such individuals frame their goals as oughts, duties, and responsibilities [7].

Overall, the RFT states that regulatory foci influence the selection and pursuit of goals, which is reflected in the desired end states that people value and in their preferred goal pursuit strategies and tactics. Specifically, the RFT distinguishes between growth versus safety goals, advancement versus maintenance goals, and ideals versus oughts. When a goal aligns with one’s regulatory focus, the person is more likely to perceive and understand the consequences of success or failure in achieving this goal [21] and is more committed to it [22,23]. For example, consumers pay more attention to different aspects of products depending on their individual regulatory focus: prevention-focused persons are more concerned with safety- or reliability-oriented features, while promotion-focused persons are concentrated more on comfort- or advancement-oriented qualities [24–26]. Prevention-oriented individuals choose goals that require maintenance (stability), while promotion-oriented individuals prefer change [10]. Research has also shown that—due to safety orientation—prevention is related to a greater likelihood of engaging in health-protective goals and activities (e.g., vaccination; [27]) and is linked with lower levels of financial risk tolerance [28]. On the other hand, people with promotion concerns want to maximize gains, so they choose goals that are highly valuable and have a high likelihood of attainment [29]. Work psychology research has demonstrated that individuals report higher justice perceptions and lower burnout when they work in an organization whose values and goals are congruent with their individual regulatory focus [30]. Moreover, when a goal pursuit tactic is consistent with a regulatory focus, a person is more confident in his or her decision and derives more psychological benefit from such decisions [31].

In this project, we postulate that the preferences for growth versus safety, advancement versus maintenance, and ideals versus oughts that arise from regulatory foci concerns could translate into the value that people place on aims that go beyond their personal goals. More specifically, the premises of the RFT can be used to explain which directions citizens consider important for their country to take. For instance, the directions that involve maximizing gains or advancing opportunities are potentially congruent with promotion and therefore could be valued more by promotion-oriented participants. On the other hand, goals that underline establishing safety or ensuring against deterioration of a given situation could be more attractive to prevention-oriented individuals.

### Problem and goals

The main claim of this article is that regulatory foci are proximal predictors of people’s preferences for the direction they think their society should take. The overarching research question is whether individual differences in regulatory foci (promotion and prevention) are related to preferences for the direction of societal development. To answer this question, two consecutive studies have been designed. In the first study, we will investigate whether different paths of societal development can be clustered with respect to regulatory focus concerns. Specifically, RFT experts will analyze a list of societal development directions and assess the extent to which each represents a promotion and prevention goal. This step will enhance understanding of the societal development direction in which promotion predominates over prevention, in which prevention predominates over promotion, or in which these dimensions are both high, or both are low.

The first assignments made by the experts will be supported by the findings of a correlational study conducted with representative samples from two countries differing in dominating regulatory focus, namely, Poland and the USA (Study 2). In this study, individual promotion and prevention orientations will be examined to determine whether they are predictors of the perceived importance of the specific societal development directions that were identified as promotion and/or prevention goals by the experts in Study 1.

## Study 1

For this project, we assume that the various paths of societal development can be divided into relatively homogenous groups in terms of whether they represent different levels of promotion and/or prevention concerns. Hence, the main aim of Study 1 is to match the potential societal development directions with regulatory foci. To this aim, a concept group mapping study will be conducted in which experts in the field of RFT will map societal developmental goals and group these goals in terms of the degree to which they represent a promotion and/or prevention focus.

### Method

#### Participant recruitment and inclusion/exclusion criteria

The snowball method will be used to recruit at least thirty experts in RFT to participate in a three-part online study [32]. Renowned scholars with a publication record in RFT will be approached by email by the principal investigator of this project and invited to participate in the study. Furthermore, they will be asked to nominate other colleagues whom they consider experts in RFT.

Individual communication to potential participants will include a description of the purpose, phases, and time frame of the study, accompanied by a link to a preliminary survey. The preliminary survey will collect the following data: name, academic affiliation, academic position, and research experience in RFT (number of publications on RTF). These data will be stored separately from participants’ email addresses to ensure participant anonymity.

After this initial screening, participants who declare that they have not authored or co-authored any publications on RFT will be excluded. Furthermore, participants will be excluded who (a) rated the societal development directions with respect to promotion and prevention using the same response for all items (zero variance); or (b) grouped societal development directions in nondisjunctive clusters, or (c) did not include one or several societal development directions in the cluster solution.

#### Study design, procedures and measures

Group Concept Mapping (GCM) will be used, which is a structured method for grouping topics, ideas, or features of certain objects according to given criteria [32,33]. GCM combines qualitative and quantitative approaches to collecting and analyzing data. Study 1 will consist of four steps: (1) assessing the extent to which societal development directions represent promotion and prevention; (2) clustering of the societal development directions presented on a two-dimensional map; (3) providing comments on the average clustering maps; and (4) accepting the final solution. At the beginning of each step, the respondents will receive information about the objective of the study, data controller, principal investigator, and data processors, the study procedure, the benefits and risks associated with the study, and the type of data collected. In the last part of each step, participants will have the opportunity to provide comments and remarks.

**Fig 1:**
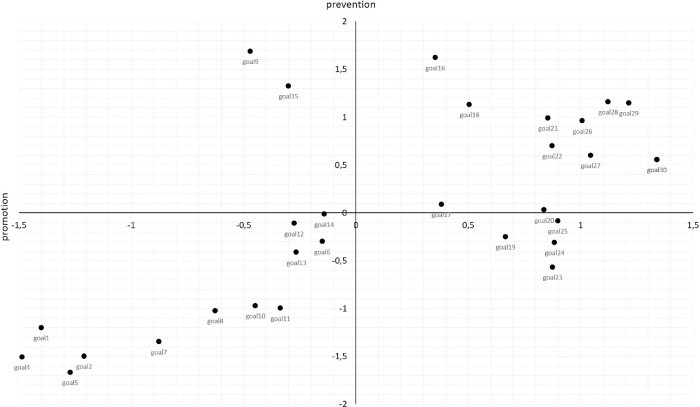
An example of a point map based on multidimensional scaling in Study 1.

The aim of step 1 will be to obtain ratings of promotion and prevention for societal development directions based on the list used in previous research by Krys et al. [5] (see S1 Appendix for the revised list of societal development goals). To this end, experts will rate the extent to which each of the societal development directions is a promotion and prevention goal using an 11-point scale ranging from 0 (*not at all)* to 10 (*very much*). The order of the aims, as well as the order of the rating (promotion, prevention), will be randomized. Based on the evaluations given by the experts, a map showing the directions of societal development in relation to two dimensions, promotion and prevention, will be prepared (see Fig 1). This map will be a graphic representation of the averaged results from this stage.

The aim of step 2 will be to obtain meaningful clusters of societal development directions on the maps. The map created in the previous step will be presented to the experts, and they will be asked to group the societal development directions shown on the maps into relatively coherent clusters (groups) according to the following criteria:

- The societal development directions should be grouped according to their similarity in the degree to which they represent promotion and prevention orientation;
- The groups should be neither too broad nor too narrow;
- The clusters must be separate/discrete, which means that each goal can only be part of one group.

**Fig 2:**
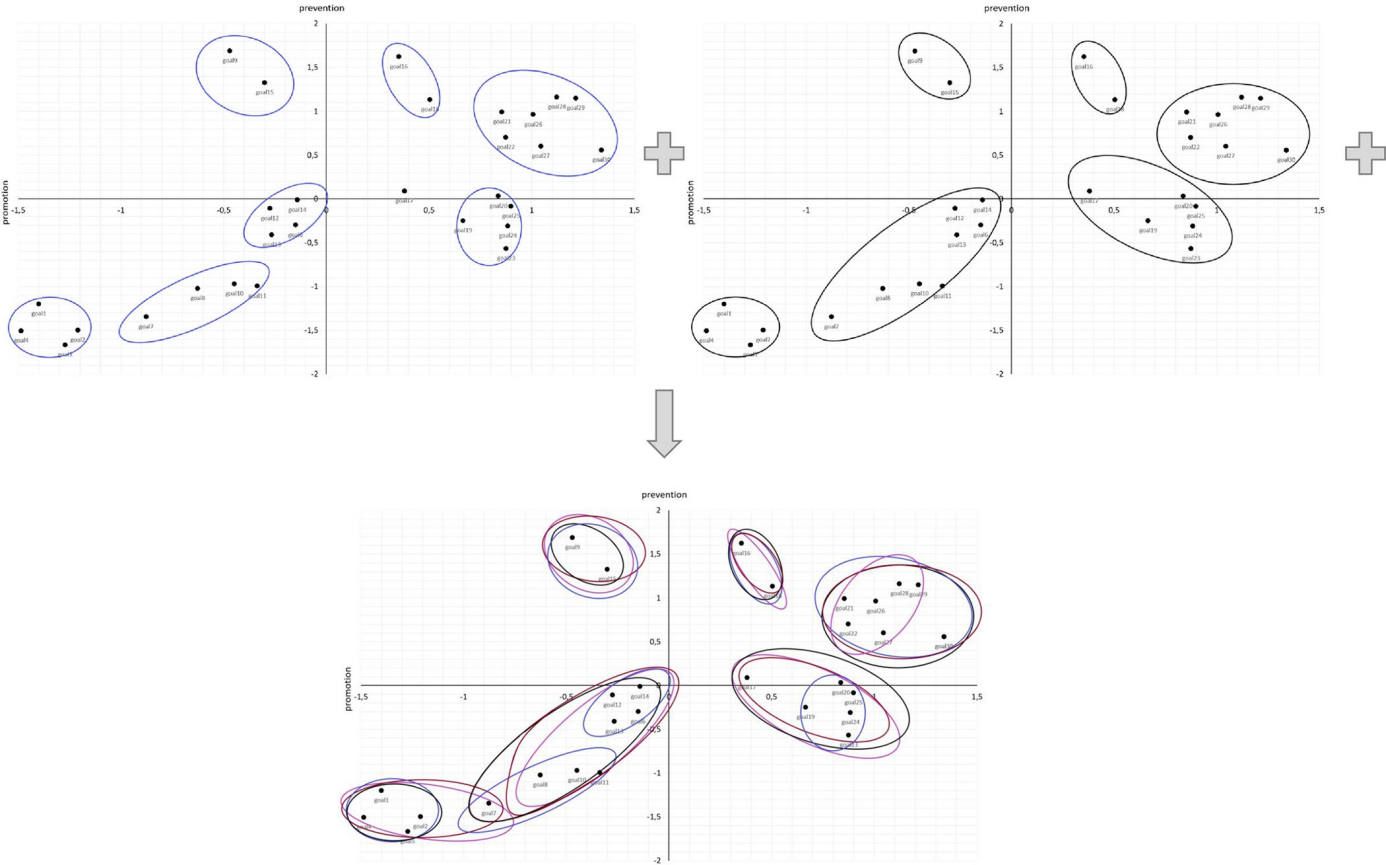
An example of possible cluster solutions in Study 1.

The aim of step 3 will be to obtain a common map with meaningful clusters of societal development directions. To this end, the experts’ suggestions of clusters will be aggregated (see Fig 2). Then, participants will be asked to analyze the common map and come up with a common solution. Finally, in step 4, this common map with clusters of societal developmental directions based on the dimensions of prevention and promotion will be presented to all experts for approval.

#### Analytical strategy

To create a map with the location of societal development directions in relation to two dimensions (promotion and prevention), the multidimensional scaling (MDS) procedure will be applied. First, for each type of assessment, an MDS with one dimension will be conducted to obtain a one-dimensional map with societal development directions ranked according to their mutual distances in promotion and prevention assessments. Second, two one-dimensional maps will be combined to obtain a map with two dimensions: promotion (X-axis) and prevention (Y-axis). Applying the MDS to the evaluations for each dimension separately and combining the results in the next step will allow us to establish the perception of societal development directions in relation to promotion and prevention dimensions. Thus, the results will be visualized using a two-dimensional scatter plot, with points representing different societal development directions.

## Study 2

The goal of Study 2 is to investigate the relationship between chronic promotion and prevention foci and the importance assigned to the specific directions of societal development. Since the exact number and characteristics of clusters of social development goals is not known, only general predictions about the relationships between the variables of interest are formulated at this point. We expect that a higher individual chronic promotion focus will be related to stronger importance placed on the country to pursue aims classified in Study 1, as related to promotion and a higher probability of choosing these aims over others. In turn, we expect that a higher individual chronic prevention focus relates to stronger importance placed on the country to pursue aims classified in Study 1, as related to prevention and a higher probability of choosing these aims over others. Specific hypotheses will be preregistered and formulated once the results of Study 1 (number and types of clusters of goals) are known. Furthermore, we expect that the relationships between regulatory foci and the importance of specific societal development goals would remain significant, controlling for covariates, that is, the perceived threat of war and subjective social status as controlled variables. We chose to control for the perceived threat of war because it is possible that there will still be a military conflict between Russia and Ukraine during data collection. Such threats may affect the relative importance of certain goals, e.g., those related to citizen security or military expansion. In addition, it has been argued that some aims, such as environmental sustainability, are considered less important in developing countries than in developed countries [34,35]. This difference might also be present within a country, just as the socioeconomic status of participants may be related to the perceived importance of their country’s goals. Hence, we also plan to control for this variable. Finally, we expect that the pattern of results would be the same in two countries differing in promotion and prevention foci, that is, Poland and the USA.

### Method

#### Participants, recruitment and inclusion/exclusion criteria

To calculate the estimated sample size for both subsamples in this study, an a-priori power analysis was conducted, assuming a significant small effect of ΔR2 = .01 in a regression analysis with a total number of four predictors: promotion and prevention foci as two predictors and the perceived threat of war and subjective social status as covariates. The analysis conducted with G*Power [36] revealed that a total sample size of 1532 per country is required to detect the expected effect with a power of 1-β = .95 and α = .05. Based on the experience of similar studies, a drop-out rate of approximately 10% (e.g., due to failed attention checks) is expected. Hence, we plan to recruit a total sample size of *N* = 1685 Polish and *N* = 1685 American adults (18 years old and higher) from a national research panel and Prolific Academic, respectively. These two sources will yield nationally representative samples and will facilitate recruitment, given the required sample size. The sample will be stratified across three demographics, age, sex, and ethnicity, as the criteria for representativeness. For the U.S. sample, Prolific will use census data from the U.S. Census Bureau to divide the sample into subgroups with the same proportions as the national population. In Poland, data from GUS (Główny Urząd Statystyczny; eng. Central Statistical Office; [37]) will be used. Individuals will be rewarded for their participation.

There will be no specific inclusion criteria. Two attention checks will be included: one in the form of an open-ended question (e.g., Please provide the current date) and the second in the form of a radio question in which they will be asked to mark a specific answer (e.g., Please answer “Not worried at all” to this question; Please answer “a small priority” to this question). Data obtained from participants who fail two attention checks implemented in the survey (questions) will be excluded [38].

#### Study design, procedures, and measures

The study will be conducted online in Polish (the Polish sample) or English (the U.S. sample). After providing informed consent, participants will be asked to complete several questionnaires presented in a random order (see measures listed below). Finally, they will be asked about their age, sex, and ethnicity, and they will be provided with feedback on the study.

### Measures

Promotion and prevention foci will be measured using two different scales: the Regulatory Focus Questionnaire (RFQ [14], Polish adaptation [39]) and the Regulatory Focus Scale (RFS [40] Polish adaptation [39]). Importantly, each of these scales measures different aspects of the RF construct [39]. The RFQ consists of two factors—the promotion and prevention factors—and the levels of these orientations are derived from past experiences. More precisely, the authors of the RFQ postulate that past experiences of success derived from promotion-linked attitudes lead to promotion pride, while past experiences of success derived from prevention-linked attitudes lead to prevention pride [40]. Thus, the items of the RFQ refer to the past—partially to childhood—and may lead to less precise answers. Examples of items for the RFQ [14] are “Compared to most people, are you typically unable to get what you want out of life?” (Promotion), and “Growing up, would you ever >crossed the line< by doing things that your parents would not tolerate?” (Prevention). In turn, the RFS comprises four factors: Openness to New Things and Autonomy, constituting the promotion dimension, and Sense of Obligation and Orientation to the Expectations of Others, forming the prevention factor. The items of the RFS relate to the present [40], with examples such as “I like trying out lots of different things, and am often successful in doing so” (Promotion) and “For me, it is very important to carry out the obligations placed on me” (Prevention). During the scale validation process in Poland, Bąk and colleagues [39] noted that only a low positive correlation (*r* = .21) was found between the prevention factors in the RFQ and RFS, and a medium positive correlation (*r* = .46) was identified between promotion factors in these two instruments. The differences in content between the RFQ and RFS and the relatively low correlations between their factors suggest that the RFQ and RFS may be measuring slightly different aspects of the construct of regulatory focus. Therefore, to capture a broader theoretical construct, it seems reasonable to combine them to achieve greater validity. Hence, in this analysis, two latent variables representing general promotion and prevention foci will be used, manifesting in the respective scales from the RFQ and RFS. In the survey, original response scales (i.e., a 5-point response scale) ranging from 1 (never or seldom) to 5 (very often) will be used for the RFQ, and a 7-point response scale ranging from 1 (definitely untrue) to 7 (definitely true) will be employed for the RFS.

To measure the importance of the direction of societal development, the same list of 31 directions of societal development as in Study 1 will be used. Participants will be asked to rate the importance of each of them using an 11-point scale, from 0 (*not important*) to 10 (*extremely important*). After that, participants will be asked to choose three aims that they find the most important. For each cluster of goals identified by the experts in Study 1, two variables will be used: the average importance of the goals within each cluster (ranging from 0 to 10) and whether this cluster includes goals indicated as the most important (nominal scale 0/1). The exact number of dependent variables for each participant will depend on the number of clusters found in Study 1.

To measure the perceived threat of war, a five-item scale developed by Wasiel et al. [41] will be used. An example of an item is “Are you worried that Russia’s invasion in Ukraine poses a real threat to the US [PL] and its allies?”. The answers will be given on a 5-point scale, with the specific numbers ranging from 1 *(not worried at all*) to 5 *(extremely worried*).

Socioeconomic status will be measured with the MacArthur Subjective SES Scale [42], which consists of a drawing of a ladder with 10 rungs representing people of different educational, income, and occupational statuses. Each rung of the ladder is assigned a number between 1 and 10, with higher numbers indicating higher placement on the ladder. Participants will be instructed to indicate a number on the rung where they feel they stand relative to their society.

#### Analytical strategy

Before testing the research hypotheses, the measurement invariance of the RFQ and RFS in their Polish and English versions will be checked using confirmatory factor analysis conducted with JASP/Lavaan or MPlus. The psychometric equivalence of the scales across the two groups with respect to configural, metric, and scalar invariance will be examined [43].

The research hypotheses will be tested with structural equation modeling using JASP/Lavaan or MPlus software and maximum likelihood with robust error estimation. First, a model with chronic promotion prevention and promotion orientations will be tested as two latent independent variables and the average importance of developmental goals within each cluster as dependent variables (assuming that these variables might be correlated) in the whole sample (pooled Polish and U.S. samples). Second, a multigroup analysis will be conducted to compare whether the pattern of results is the same in the two samples. If we find that the unconstrained model fits the data better than the constrained model, that is, the country moderates the relations between individual regulatory foci and the perceived importance of societal goals, we will further test the differences between the models fitted in the two countries with respect to specific structural paths. The same analyses for the second set of dependent variables, i.e., those based on the three goals deemed most important, will be performed.

In addition, the same models as above will be tested, controlling for the perceived threat of war and subjective socioeconomic status. These variables will be included as covariates of both the regulatory focus variables and the importance of societal development goal variables.

## Status and timeline of the study

This study protocol has been submitted before the start of all data collection. The research described in this protocol was approved by the Departmental Ethical Review Board at SWPS University of Social Sciences and Humanities (WKE/S 2022/9/VI/119). In both studies, the participants will provide written informed consent.

Data collection in Study 1 will start just after the study protocol is accepted, and data analysis will start after the data collection in stage 1 of this study is completed. Concerning Study 2, data collection will begin after the data analysis of Study 1 is completed, and it will last until the recruitment goal is fulfilled. Data analysis for Study 2 will begin after all data has been collected.

## Data management and dissemination plan

To maintain anonymity and protect the privacy of participants, all personal data (Study 1) will be stored in password-protected files and will be protected against access by unauthorized persons. No personal data will be collected in Study 2. Individual data will not be reported in any publication(s). The preprocessed and anonymized data from both studies will be stored in the Open Science Framework (OSF) repository. We plan to prepare and submit at least one paper to a leading journal in the field.

## Discussion

This project has the potential to contribute to a better understanding of the folk theories of societal development. More specifically, the project will make three important contributions to the field. First, the novel contribution of this project lies in approaching folk theories of societal development from the perspective of motivational orientations, looking at individual-level determinants of preferences that are exhibited at the country level. Previous research has demonstrated that there are cross-cultural differences in the predominance of regulatory focus [44,45], and therefore, we expect that these differences are responsible for the preference in the societal development directions that their countries might follow. In the future, this could help increase understanding, for example, of how macro-level differences in regulatory focus concerns may be associated with incongruencies in how citizens value the importance of certain societal development goals over others.

Another reason to include regulatory foci in the conceptualization of preferences for directions of societal development is that the independence of promotion and prevention foci has important implications for a more nuanced understanding of societal development goals. Specifically, these goals could be categorized into various groups that may have different appeals to citizens, for example, goals that are high on both prevention and promotion, goals that are high only on promotion or only on prevention, and goals that have no characteristics of promotion or prevention. Thus, the second contribution of this project is a better understanding of the role of dual regulatory focus, or the dominant focus for each goal, in predicting goal pursuit preferences and behaviors. In particular, this knowledge could be used to determine the types of incentives that might be effective in instigating motivation toward a particular goal.

Third, the research will allow us to analyze the conceptions of people on societal development, i.e., which directions are perceived as the “right” ones. This is relevant because such “intuitive theories” that people affect their cognition, emotions, and behaviors [46] could have an impact on their well-being. Specifically, if a particular direction is considered valuable by an individual but the country does not prioritize it, this can be detrimental to such a person’s well-being and the experience of connectedness or national identification. From the public policy perspective, reliable information on people’s preferences toward societal development pathways may also help "democratize" societal development processes.

The research proposed in this project will employ GCM to match the societal development goals with regulatory focus concerns, and these findings will be further corroborated with quantitative correlational studies performed in two countries. The strength of the design used is the combination of a qualitative and quantitative approach in GCM, which allows for a more nuanced understanding of the phenomenon studied. The GCM method is suitable for studying complex phenomena that require the inclusion of multiple perspectives [33]. In this project, experts with high levels of expertise in RFT will be recruited from different parts of the world to achieve a broader and more comprehensive view. The iterative process, in which participants both provide information and help interpret the results, will strengthen the validity of the findings.

Additionally, the original assessments and the cluster solution generated by the experts in Study 1 will be corroborated by the correlational findings in Study 2, reinforcing the accuracy of the mapping of the different pathways of societal development with regulatory focus concerns. Finally, previous research indicates that people from different cultural contexts might differ in their predominant regulatory focus: for example, Poles tend to be predominantly prevention-oriented [47], while Americans tend to be promotion-oriented [15]. Importantly, the strength of this project is that we will recruit representative samples from two countries to ensure that the patterns that we discover are not limited to a particular cultural context.

All study materials, databases, and scripts will be available in an open science repository, and the detailed hypotheses, sample size, and analyses for Study 2 will be preregistered, which will strengthen the replicability and reproducibility of this research. The empirical papers describing the results will be disseminated in open access.

## Supporting information

S1 Appendix The revised list of societal development goals. (DOCX)
