# Supplementary material for: Maximizing economic prosperity, strengthening military, or developing social bonds? Study protocol for research on the relationship between regulatory focus and preferences for the direction of societal development
Source: PLoS One. 2022 Sep 26;17(9):e0274624. doi: 10.1371/journal.pone.0274624 (PMC9512174; doi:10.1371/journal.pone.0274624)
Supplement: S1 Appendix — (DOCX) [file pone.0274624.s001.docx]

S1 Appendix

List of societal development aims that will be used in Study 1 and Study 2

1. Getting rid of poverty
2. Making life easier for families
3. Emphasizing religious values
4. Being accepting of people coming to live in (country name) from other countries
5. People living healthy long lives
6. Living in freedom
7. Having strong military power
8. Fighting all forms of inequality
9. Providing high quality education
10. Maximizing economic prosperity
11. Increasing birth rates
12. Building trust between people
13. Protecting human rights
14. Having a well-functioning democracy
15. Strengthening social ties among people
16. Protecting the environment
17. Developing industry
18. Enabling people to work and study remotely
19. Fighting inequality between men and women
20. Keeping stable prices
21. Investing in science
22. Manufacturing more
23. Providing common access to fast internet
24. Fighting corruption
25. Having a well-functioning justice system
26. Strengthening the country currency
27. Supporting the development of companies
28. Opening more factories
29. Making people feel safe in the streets
30. Enabling people to complete official procedures on-line
31. Emphasizing (country name) traditions
